# Supplementary material for: Risk factors for disease severity and increased medical resource utilization in respiratory syncytial virus (+) hospitalized children: A descriptive study conducted in four Belgian hospitals
Source: PLoS One. 2022 Jun 6;17(6):e0268532. doi: 10.1371/journal.pone.0268532 (PMC9170098; doi:10.1371/journal.pone.0268532)
Supplement: S1 File — (ZIP) [file pone.0268532.s001.zip › Supplementary section files_24Mar22/List of supplementary tables and figures.pdf]

Supporting information:

S 1: Physical Examination Scoring

S 2: Baseline characteristics as per age, symptom length and underlying risk

S 3: Medical Resource Utilization during the study as per age group, symptom length and underlying risk

S 4: PES item score over time as per age

S 5: PES item score over time as per symptom onset

S 6: Length of hospitalization by age and underlying risk

S 7: Logistic Regression analysis for length of hospital stay >4 days

S 8: Cox proportional hazard regression analysis for length of hospital stay

S 9: Logistic Regression analysis for probability of receiving oxygen supplementation
