# Supplementary material for: Risk factors for disease severity and increased medical resource utilization in respiratory syncytial virus (+) hospitalized children: A descriptive study conducted in four Belgian hospitals
Source: PLoS One. 2022 Jun 6;17(6):e0268532. doi: 10.1371/journal.pone.0268532 (PMC9170098; doi:10.1371/journal.pone.0268532)
Supplement: S1 File — (ZIP) [file pone.0268532.s001.zip › Supplementary section files_24Mar22/S 1.pdf]

Supplemental Digital Content 1: Physical Examination Scoring

**Level**	**0**	**1**	**2**	**3**
**Ability to feed**	normal		Reduced	Intravenous fluid or nasogastric tube feeding
**Otitis**	None	Dulled tympanic membrane	Inflamed, infected tympanic membrane	Retracted or bulging tympanic membrane, obvious air-fluid level
**Cough**	No	Just noticeable	Bothersome sometimes, not interfering with feeding	Bothersome most of the time, interfering with feeding
**Nasal Discharge**	None	Clear, slightly increased	Clear to white, obvious increased volume	Frankly purulent (yellow or green), or gross blood
**Dyspnea**	None	May have brief episodes	May have increased episodes	May have severe episodes
**Respiratory Effort**	None	Mild chest wall retraction	Trachea tug with moderate chest wall retraction and nasal flare	Marked chest wall retraction with nasal flare
**Rales, rhonchi or other**	None	No Level 1	Scattered wheezes or rhonchi	Widespread wheezes or rhonchi, rales, dyspnea, or signs of consolidation
**Wheezing**	None	Terminal expiration or only with stethoscope	Entire expiration or audible on expiration without stethoscope	Inspiration and expiration audible without stethoscope
