# Supplementary material for: Risk factors for disease severity and increased medical resource utilization in respiratory syncytial virus (+) hospitalized children: A descriptive study conducted in four Belgian hospitals
Source: PLoS One. 2022 Jun 6;17(6):e0268532. doi: 10.1371/journal.pone.0268532 (PMC9170098; doi:10.1371/journal.pone.0268532)
Supplement: S1 File — (ZIP) [file pone.0268532.s001.zip › Supplementary section files_24Mar22/S 3.pdf]

Supplemental Digital Content 3. Medical Resource Utilization during the study as per age group, symptom length and underlying risk

Parameter	Age[a]				Symptom length		Underlying Risk	
	0–<3 months (n = 28)	3–<6 months (n = 15)	6–<12 months (n = 13)	12–48 months (n = 18)	≤3 days (n = 43)	>3 days (n = 32)	Yes (n = 14)	No (n = 61)
**Co-medication during study (n [%])**								
Antibiotics	6 (21.4)	7 (46.7)	6 (46.2)	12 (66.7)	20 (46.5)	11 (34.4)	10 (71.4)	21 (34.4)
Bronchodilators	10 (35.7)	12 (80.0)	11 (84.6)	14 (77.8)	24 (55.8)	24 (75.0)	9 (64.3)	39 (63.9)
Corticosteroids	1 (3.6)	0	2 (15.4)	3 (16.7)	3 (7.0)	3 (9.4)	2 (14.3)	4 (6.6)
Others[b]	1 (3.6)	1 (6.7)	0	2 (11.1)	2 (4.7)	2 (6.3)	0	4 (6.6)
**Length of hospital stay (days, median [range])[c]**	5.0 (2–7)	4.5 (2–7)	6.0 (2–7)	4.0 (2–6)	4.0 (2–7)	5.0 (2–7)	4.0 (2–7)	5.0 (2–7)
**Length of hospital stay (days, n [%])**								
2	3 (10.7)	1 (6.7)	1 (7.7)	2 (11.1)	2 (4.8)	5 (15.6)	1 (7.1)	6 (10.0)
3	3 (10.7)	4 (26.7)	1 (7.7)	3 (16.7)	6 (14.3)	5 (15.6)	2 (14.3)	9 (15.0)
4	4 (14.3)	2 (13.3)	1 (7.7)	4 (22.2)	10 (23.8)	1 (3.1)	4 (28.6)	7 (11.7)

5	5 (17.9)	1 (6.7)	2 (15.4)	4 (22.2)	6 (14.3)	6 (18.8)	1 (7.1)	11 (18.3)
6	4 (14.3)	5 (33.3)	2 (15.4)	2 (11.1)	6 (14.3)	7 (21.9)	2 (14.3)	11 (18.3)
7	2 (7.1)	1 (6.7)	4 (30.8)	0	2 (4.8)	5 (15.6)	1 (7.1)	6 (10.0)
>7	5 (17.9)	1 (6.7)	1 (7.7)	1 (5.6)	6 (14.3)	2 (6.3)	2 (14.3)	6 (10.0)
**Oxygen supplementation (n [%])**								
Yes	23 (82.1)	7 (46.7)	7 (53.8)	7 (38.9)	25 (58.1)	19 (59.4)	5 (35.7)	39 (63.9)
No	5 (17.9)	8 (53.3)	6 (46.2)	11 (61.1)	18 (41.9)	13 (40.6)	9 (64.3)	22 (36.1)
**Length of oxygen supplementation (days, median [range])**[d]	4.0 (1–7)	2.0 (1–4)	3.0 (1–5)	3.0 (2–5)	3.0 (1–7)	3.0 (1–6)	2.0 (2–5)	3.0 (1–7)
**Length of oxygen supplementation (days, n [%])**[d]								
1	1 (4.3)	2 (28.6)	1 (14.3)	0	2 (8.0)	2 (10.5)	0	4 (10.3)
2	5 (21.7)	2 (28.6)	2 (28.6)	3 (42.9)	9 (36.0)	3 (15.8)	3 (60.0)	9 (23.1)
3	5 (21.7)	1 (14.3)	2 (28.6)	3 (42.9)	5 (20.0)	6 (31.6)	0	11 (28.2%)

4	5 (21.7)	2 (28.6)	1 (14.3)	0	4 (16.0)	4 (21.1)	1 (20.0)	7 (17.9)
5	3 (13.0)	0	1 (14.3)	1 (14.3)	2 (8.0)	3 (15.8)	1 (20.0)	4 (10.3)
6	2 (8.7)	0	0	0	1 (4.0)	1 (5.3)	0	2 (5.1)
7	2 (8.7)	0	0	0	2 (8.0)	0	0	2 (5.1)
**Visited family doctor for ARI before hospitalization (n [%])**								
Yes	13 (46.4)	11 (73.3)	3 (23.1)	11 (61.1)	17 (39.5)	22 (68.8)	5 (35.7)	34 (55.7)
No	15 (53.6)	4 (26.7)	10 (76.9)	7 (38.9)	26 (60.5)	10 (31.3)	9 (64.3)	27 (44.3)

[a] The sample size for the evaluation was 74 since data was missing for 1 patient.

[b]One patient received Synagis (older age group, likely immunocompromised and presented early [≤3d]).

[c] The sample sizes for this evaluation were 21, 14, 11 and 15 for 0–<3 months, 3–<6 months, 6–<12 months and 12–48 months groups, respectively; 30 and 25 for ≤3 days and >3 days intercept groups, respectively; and 11 and 50 for yes and no underlying risk groups, respectively.

[d] The sample sizes for this evaluation were 23, 7, 7, and 7 for 0–<3 months, 3–<6 months, 6–<12 months and 12–48 months groups, respectively; 25 and 18 for ≤3 days and >3 days intercept groups, respectively; and 5 and 39 for yes and no underlying risk groups, respectively.

1 subject with censored length of hospital stay >3 days, 2 subjects with censored length of hospital stay >4 days, and 2 subjects with censored length of hospital stay >5 days were not presented.

Abbreviations: ARI – Acute Respiratory Infection
