# Supplementary material for: Risk factors for disease severity and increased medical resource utilization in respiratory syncytial virus (+) hospitalized children: A descriptive study conducted in four Belgian hospitals
Source: PLoS One. 2022 Jun 6;17(6):e0268532. doi: 10.1371/journal.pone.0268532 (PMC9170098; doi:10.1371/journal.pone.0268532)
Supplement: S1 File — (ZIP) [file pone.0268532.s001.zip › Supplementary section files_24Mar22/S 5.pdf]

Supplemental Digital Content 5: PES item score over time as per symptom onset

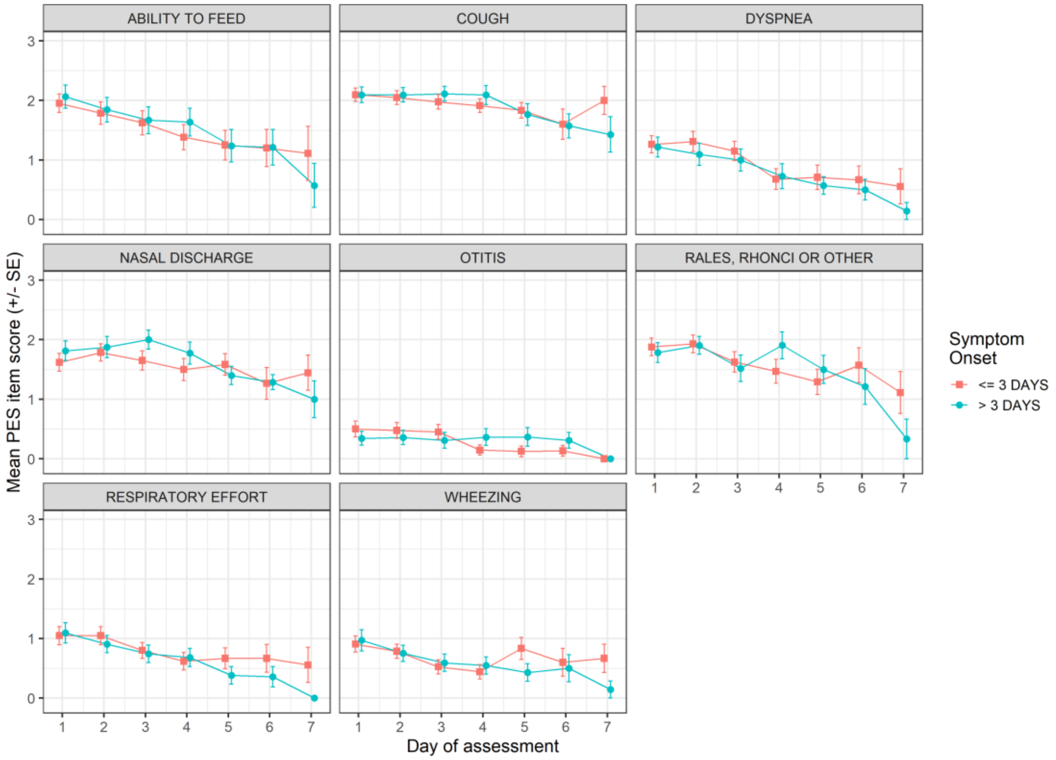

Mean ± SE PES item score is represented at each day of assessment for patient classified based on symptom onset or intercept.

Abbreviations: PES – Physical Examination Scoring, SE – Standard Error
