# Supplementary material for: Risk factors for disease severity and increased medical resource utilization in respiratory syncytial virus (+) hospitalized children: A descriptive study conducted in four Belgian hospitals
Source: PLoS One. 2022 Jun 6;17(6):e0268532. doi: 10.1371/journal.pone.0268532 (PMC9170098; doi:10.1371/journal.pone.0268532)
Supplement: S1 File — (ZIP) [file pone.0268532.s001.zip › Supplementary section files_24Mar22/S 7.pdf]

Supplemental Digital Content 7: Logistic Regression analysis for length of hospital stay >4 days

Parameter	Univariate analysis		Multivariate analysis	
	OR (95% CI)	p value[a]	OR (95% CI)	p value[a]
**Age**				
0–<3 months	-	0.455		0.402
3–6 months	0.57 (0.15–2.10)		0.53 (0.11–2.29)	
6–<12 months	1.67 (0.39–8.82)		1.73 (0.34–10.81)	
12–<48 months	0.56 (0.16–1.96)		0.53 (0.12–2.23)	
**Gender**				
Female	-	0.542	-	0.418
Male	1.35 (0.52–3.54)		1.51 (0.56–4.23)	
**Underlying risk**				
No	-	0.288	-	0.315
Yes	0.52 (0.16–1.74)		0.50 (0.13–1.95)	
**Length of symptoms at intercept**				
≤3 days	-	0.624	-	0.762
>3 days	1.27 (0.49–3.40)		1.19 (0.39–3.70)	
**Oxygen supplementation at day 1**				
No	-	0.758	-	0.645
Yes	1.16 (0.44–3.11)		0.76 (0.24–2.40)	
**PES Score - Feeding**				

Score 0	-	0.768		
Score 2	0.89 (0.23–3.27)			
Score 3	1.33 (0.32–5.40)			
**PES Score - Dyspnea**				
Score 0	-			
Score 1	1.42 (0.41–4.98)	0.854		
Score 2	1.71 (0.47–6.42)			
Score 3	1.07 (0.18–6.78)			
**PES Score – Respiratory effort**				
Score 0	-	0.923		
Score 1	0.81 (0.25–2.52)			
Score 2	1.29 (0.30–5.99)			
Score 3	0.95 (0.18–5.64)			
**PES3-total score (3 items; 1-unit increase)**	1.05 (0.85–1.31)	0.658	1.12 (0.88–1.42)	0.353

N=72, 1 patient was excluded from analysis as information on length of stay and PES score on day 1 was unavailable. 2 patients were excluded as they did not have PES score on day 1 available. The patient with censoring value >3 days was considered as having a length of stay >4 days.

[a]p value was calculated by a likelihood ratio test.

Abbreviations: CI – Confidence Interval, OR – Odds Ratio, PES – Physical Examination Scoring, PES3 – PES sum of individual scores for feeding, dyspnea, respiratory effort
