# Supplementary material for: Risk factors for disease severity and increased medical resource utilization in respiratory syncytial virus (+) hospitalized children: A descriptive study conducted in four Belgian hospitals
Source: PLoS One. 2022 Jun 6;17(6):e0268532. doi: 10.1371/journal.pone.0268532 (PMC9170098; doi:10.1371/journal.pone.0268532)
Supplement: S1 File — (ZIP) [file pone.0268532.s001.zip › Supplementary section files_24Mar22/S 9.pdf]

Supplemental Digital Content 9: Logistic Regression analysis for probability of receiving oxygen supplementation

Parameter	Univariate analysis		Multivariate analysis	
	OR (95% CI)	p value[a]	OR (95% CI)	p value[a]
**Age**				
0–<3 months	-	0.003	-	0.005
3–6 months	0.15 (0.03–0.63)		0.09 (0.01–0.49)	
6–<12 months	0.20 (0.04–0.90)		0.17 (0.02–0.98)	
12–<48 months	0.09 (0.02–0.38)		0.07 (0.01–0.36)	
**Gender**				
Female	-	0.883	-	0.669
Male	1.07 (0.42–2.77)		1.29 (0.41–4.22)	
**Underlying risk**				
No	-	0.043	-	0.129
Yes	0.29 (0.08–0.96)		0.29 (0.05–1.43)	
**Length of symptoms at intercept**				
≤3 days	-	0.957	-	0.739
>3 days	0.97 (0.38–2.53)		1.24 (0.35–4.45)	
**PES Score - Feeding**				
Score 0	-	0.812		
Score 2	0.79 (0.20–2.88)			
Score 3	1.11 (0.27–4.43)			
**PES Score - Dyspnea**				

Score 0	-	0.062		
Score 1	3.56 (1.03–13.49)			
Score 2	5.33 (1.44–22.31)			
Score 3	5.00 (0.82–42.97)			
**PES Score – Respiratory effort**				
Score 0	-	0.086		
Score 1	2.74 (0.89–8.91)			
Score 2	2.31 (0.58–9.97)			
Score 3	10.11 (1.44–206)			
**PES3-total score (3 items; 1-unit increase)**	1.32 (1.04–1.73)	0.023	1.63 (1.19–2.37)	0.002

N=72, 2 patients were excluded as they did not have PES score on day 1 available. 1 patient was excluded due to missing age.

[a]p value was calculated by a likelihood ratio test.

Abbreviations: CI – Confidence Interval, OR – Odds Ratio, PES – Physical Examination Scoring, PES3 – PES sum of individual scores for feeding, dyspnea, respiratory effort
