# Supplementary material for: Risk factors for disease severity and increased medical resource utilization in respiratory syncytial virus (+) hospitalized children: A descriptive study conducted in four Belgian hospitals
Source: PLoS One. 2022 Jun 6;17(6):e0268532. doi: 10.1371/journal.pone.0268532 (PMC9170098; doi:10.1371/journal.pone.0268532)
Supplement: S1 File — (ZIP) [file pone.0268532.s001.zip › Supplementary section files_24Mar22/S-2.pdf]

Supplemental Digital Content 2: Baseline characteristics as per age, symptom length and underlying risk

Parameter	Age				Symptom length		Underlying risk	
	0–<3 months (n = 28)	3–<6 months (n = 15)	6–<12 months (n = 13)	12–48 months (n = 18)	≤3 days (n = 43)	>3 days (n = 32)	Yes (n = 14)	No (n = 61)
**Age (months, median [range])**	1.0 (0–2)	3.0 (3–5)	8.0 (6–11)	23.0 (12–41)	2.5 (0–41)	5.5 (0–40)	8.0 (1.0–32)	3.0 (0–41.0)
**Age (months, n [%])**								
0 - <3	28 (100)	0	0	0	21 (50.0)	7 (21.9)	2 (14.3)	26 (43.3)
3 - <6	0	15 (100)	0	0	6 (14.3)	9 (28.1)	4 (28.6)	11 (18.3)
6 - <12	0	0	13 (100)	0	5 (11.9)	8 (25.0)	3 (21.4)	10 (16.7)
12 - 48	0	0	0	18 (100)	10 (23.8)	8 (25.0)	5 (35.7)	13 (21.7)
**Gender (n [%])**								
Female	12 (42.9)	7 (46.7)	7 (53.8)	8 (44.4)	20 (46.5)	14 (43.8)	6 (42.9)	28 (45.9)
Male	16 (57.1)	8 (53.3)	6 (46.2)	10 (55.6)	23 (53.5)	18 (56.3)	8 (57.1)	33 (54.1)
**RSV subtype (n [%])**								
A	26 (92.9)	14 (93.3)	12 (92.3)	16 (88.9)	38 (88.4)	30 (93.8)	13 (92.9)	55 (90.2)
B	2 (7.1)	1 (6.7)	1 (7.7)	2 (11.1%)	5 (11.6)	2 (6.3)	1 (7.1)	6 (9.8)

**Weight at birth (kg, median [range])**	3.64 (1.84–4.37)	3.15 (2.37–4.31)	3.02 (2.41–3.85)	3.10 (1.00–4.26)	3.42 (1.00–4.37)	3.10 (1.25–4.31)	3.11 (1.25–4.15)	3.44 (1.00–4.37)
**Baseline weight (kg, median [range])**	4.81 (2.68–9.90)	6.60 (5.55–12.00)	8.09 (6.34–11.10)	10.35 (8.00–17.70)	6.4 (2.74–16.70)	7.44 (2.68–17.70)	8.56 (4.80–13.00)	6.32 (2.68–17.70)
**Day care attendance (n [%])**								
Yes	2 (7.4)	7 (46.7)	12 (92.3)	14 (77.8)	18 (42.9)	18 (56.3)	9 (64.3)	27 (45.0)
No	25 (92.6)	8 (53.3)	1 (7.7)	4 (22.2)	24 (57.1)	14 (43.8)	5 (35.7)	33 (55.0)
**Currently breastfed (n [%])**								
Yes	20 (71.4)	3 (20.0)	4 (30.8)	0	18 (41.9)	9 (28.1)	4 (28.6)	23 (37.7)
No	8 (28.6)	12 (80.0)	9 (69.2)	18 (100)	25 (58.1)	23 (71.9)	10 (71.4)	38 (62.3)
**Underlying risk * (n [%])**								
Yes	2 (7.1)	4 (26.7)	3 (23.1)	5 (27.8)	11 (25.6)	3 (9.4)	14 (100)	0
No	26 (92.9)	11 (73.3)	10 (76.9)	13 (72.2)	32 (74.4)	29 (90.6)	0	61 (100)
**Premature birth (n [%])**								
Yes	3 (10.7)	3 (20.0)	4 (30.8)	4 (22.2)	7 (16.3)	8 (25.0)	3 (21.4)	12 (19.7)

No	25 (89.3)	12 (80.0)	9 (69.2)	14 (77.8)	36 (83.7)	24 (75.0)	11 (78.6)	49 (80.3)
**Symptom length at hospitalization (days, median [range])**	3.0 (1.0–5.0)	4.0 (1.0–5.0)	4.0 (1.0–5.0)	3.0 (1.0–5.0)	2.0 (1–3)	4.0 (4–5)	2.5 (1–5)	3.0 (1–5)
**Symptom length (days, n [%])**								
≤3	21 (75.0)	6 (40.0)	5 (38.5)	10 (55.6)	43 (100)	0	11 (78.6)	32 (52.5)
>3	7 (25.0)	9 (60.0)	8 (61.5)	8 (44.4)	0	32 (100)	3 (21.4)	29 (47.5)

*Underlying risk includes asthma/atopy (n=2), hyperreactive lung (n=5), CHD (n=4), immunodeficiency (n=1) and others (n=4).

Abbreviations: CHD – Congenital Heart Disease, RSV – Respiratory Syncytial Virus
