# Supplementary material for: Risk factors for disease severity and increased medical resource utilization in respiratory syncytial virus (+) hospitalized children: A descriptive study conducted in four Belgian hospitals
Source: PLoS One. 2022 Jun 6;17(6):e0268532. doi: 10.1371/journal.pone.0268532 (PMC9170098; doi:10.1371/journal.pone.0268532)
Supplement: S1 File — (ZIP) [file pone.0268532.s001.zip › Supplementary section files_24Mar22/S-8.pdf]

Supplemental Digital Content 8: Cox proportional hazard regression analysis for length of hospital stay

Parameter	Multivariate analysis	
	HR (95% CI)	p value[a]
**Age**		
0–<3 months	-	0.290
3–6 months	1.51 (0.68–3.35)	
6–<12 months	0.90 (0.38–2.13)	
12–<48 months	1.81 (0.80–4.11)	
**Gender**		
Female	-	0.573
Male	0.86 (0.50-1.47)	
**Underlying risk**		
No	-	0.913
Yes	0.96 (0.43-2.11)	
**Length of symptoms at intercept**		
≤3 days	-	0.473
>3 days	1.25 (0.68-2.28)	
**Oxygen supplementation at day 1**		
No		0.951
Yes	1.02 (0.56-1.86)	
**PES total score (3 items; 1-unit increase)**	0.94 (0.83-1.06)	0.325

N=72, 1 patient was excluded from analysis as information on length of stay and PES score on day 1 was unavailable. 2 patients were excluded as they did not have PES score on day 1 available.
[a]p value was calculated by a likelihood ratio test.
A global chi-square test for the proportional hazard assumption showed no deviation (p=0. 780). A graphical review of possible time-dependent coefficients over time (plots of residuals for individual predictors) showed no deviation from the proportional hazard assumption.
Abbreviations: CI – Confidence Interval, HR – Hazard Ratio, PES – Physical Examination Scoring
