# Supplementary material for: Risk factors for disease severity and increased medical resource utilization in respiratory syncytial virus (+) hospitalized children: A descriptive study conducted in four Belgian hospitals
Source: PLoS One. 2022 Jun 6;17(6):e0268532. doi: 10.1371/journal.pone.0268532 (PMC9170098; doi:10.1371/journal.pone.0268532)
Supplement: Attachment — Submitted filename: Comments to Authors.docx [file pone.0268532.s002.docx]

Comments to Authors:

The study demonstrates the issue well in disease severity and medical resource utilization of hospitalized RSV-positive children. Overall, this is a well-written manuscript and contributes valuable respiratory data in pediatric clinical practice. The methods are generally appropriate, although authors should clarify a few details and provide a rationale for using analytical methods to measure medical resource utilization parameters.

Introduction

p.9, paragraph 3: Since the authors chose the age as a parameter for medical resource utilization categorized into 0-<3 months, 3-6 months, 6-<12 months, 12-<48 months, readers would want to see background epidemiological data of RSV in these age groups of children.

Methods

Study design

p.11, lines 93-95: Please explain on what grounds these hospitals were selected. Are these hospitals located in RSV endemic areas in the country?

p.11, line 96: Since authors addressed epidemic seasons in the Introduction of the study, the S1 figure is unnecessary. Instead, please add study start and end dates for each respective year.

Study population

p.12, line 105: Please give more specifics of study subjects. Are all pediatric patients included who met inclusion criteria? Readers would want to know why the recruited subjects are only 75 when the RSV detection rate is almost 80% of children under five with severe acute respiratory infection in Belgium generally. (Subissi, L., Bossuyt, N., Reynders, M., Gérard, M., Dauby, N., Bourgeois, M., ... & Barbezange, C. (2020). Capturing respiratory syncytial virus season in Belgium using the influenza severe acute respiratory infection surveillance network, season 2018/19. *Eurosurveillance*, *25*(39), 1900627.)

Demographics and clinical characteristics

p.12, line 118: Did authors consider including re-infection of RSV in underlying conditions?

Medical resource utilization

p.13, line 138: As indications of medical resource utilization might differ depending on the country or context, please specify indications for supplementary oxygen provided for patients involved in the study. Also, authors might need to consider this indication as one of the covariates in the analysis if the practice differs across hospitals. Supplementary oxygen is a highly vulnerable treatment depending on the capacity of the hospitals, especially in developing countries.

Statistical analysis

p.13, line 147 and line 152: Please briefly explain why Kaplan-Meier survival analysis and Cox proportional hazard regression models were performed with distinction. Please indicate all the variables included in each analysis respectively.

p. 14, line 163: Please explain why p-values without multiple comparison corrections were reported.

Results

p. 15, line 170: Readers would want to know why children above 41 months did not take part in the study as inclusion criteria were pediatric patients up to 5 years old.

Table 1

Please clarify breastfeeding? Does it indicate breastfeeding at the point of the data collection?

Clinical disease kinetics

p.19, line 231-233: As shown in Fig 2, the ability to feed and wheezing were mentioned, but how about rales, rhonchi, or others as it appears to be presented more in patients with underlying risk factors too?

Discussion

p.23, paragraph 4: In case authors do not have data on bacterial co-infection, it might look misguiding to state that 41.3% of the patients prescribed with antibiotics highlight overuse of antibiotics. If the authors have more explanation in this regard, please do so. Otherwise, please revise.

p.24, line 330: Please discuss more on the limitation of the study, including potential sources of bias.

Minor comments:

Please correct typos such as:

p. 7, line 45: “PSE3” to “PES3”

p.8, line 59: “score” to “score.”

p.26, line 362: “children” to “children.”
