# Supplementary material for: Risk factors for disease severity and increased medical resource utilization in respiratory syncytial virus (+) hospitalized children: A descriptive study conducted in four Belgian hospitals
Source: PLoS One. 2022 Jun 6;17(6):e0268532. doi: 10.1371/journal.pone.0268532 (PMC9170098; doi:10.1371/journal.pone.0268532)
Supplement: Attachment — Submitted filename: Response to Reviewers_PONE-D-21-25349.docx [file pone.0268532.s003.docx]

November 30, 2021

Emily Chenette,

Editor-in-Chief

PLOS One

**RE:** [PONE-D-21-25349] - [EMID:89282667a1cb0907]

**Title:** Risk factors for disease severity and increased medical resource utilization in respiratory syncytial virus (+) hospitalized children: a descriptive study conducted in four Belgian hospitals

**Authors:** Marijke Proesmans, Annabel Rector, Els Keyaerts, Yannick Vandendijck, Francois Vermeulen, Kate Sauer, Marijke Reynders, Ann Verschelde, Wim Laffut, Kristien Garmyn, Roman Fleischhackl, Jacques Bollekens, Gabriela Ispas

Dear Emily,

We thank the reviewers for their valuable suggestions that have enhanced the scientific value and readability of our manuscript. Point-by-point responses to these comments are provided below with an indication of where the changes have been made in the text. All changes to the manuscript are marked as track changes, a clean copy has also been uploaded as suggested. Our revisions address each issue raised by the reviewers, and we hope that you will consider our revised manuscript suitable for publication.

Kindly contact me at the email address provided below with any queries you may have regarding the manuscript.

Sincerely,

**Gabriela Ispas**
Email: [gispas@its.jnj.com](mailto:gispas@its.jnj.com)

**Reviewers' Comments:**

1. Is the manuscript technically sound, and do the data support the conclusions?

The manuscript must describe a technically sound piece of scientific research with data that supports the conclusions. Experiments must have been conducted rigorously, with appropriate controls, replication, and sample sizes. The conclusions must be drawn appropriately based on the data presented.

Reviewer #1: Yes

Reviewer #2: Yes

________________________________________

2. Has the statistical analysis been performed appropriately and rigorously?

Reviewer #1: Yes

Reviewer #2: Yes

________________________________________

3. Have the authors made all data underlying the findings in their manuscript fully available?

The PLOS Data policy requires authors to make all data underlying the findings described in their manuscript fully available without restriction, with rare exception (please refer to the Data Availability Statement in the manuscript PDF file). The data should be provided as part of the manuscript or its supporting information or deposited to a public repository. For example, in addition to summary statistics, the data points behind means, medians and variance measures should be available. If there are restrictions on publicly sharing data—e.g. participant privacy or use of data from a third party—those must be specified.

Reviewer #1: Yes

Reviewer #2: Yes

________________________________________

4. Is the manuscript presented in an intelligible fashion and written in standard English?

PLOS ONE does not copyedit accepted manuscripts, so the language in submitted articles must be clear, correct, and unambiguous. Any typographical or grammatical errors should be corrected at revision, so please note any specific errors here.

Reviewer #1: No

Reviewer #2: Yes

________________________________________

**Reviewer 1:**

***We appreciate your thoughtful review of the manuscript and the responses to your comments are provided below***

**Comment 1:** Proesmans et al., report relevant data on risk factors for hospitalization and use of medical resources in children <5 years old in Belgium. The study nevertheless requires reorganization, better consistency, and faithful presentation of the dependent and independent variables in the methodology and results sections.

**Response:** Based on your comments, we have made the necessary changes and hope the manuscript meets your expectations

**Comment 2:** Methodology - L97: In general, it is preferred to describe the following information: (company name, City, State. Country) for company information of the experimental kit used.

**Response:** Either the standard of care quantitative RT-PCR (qRT-PCR) test which concerns a semi-quantitative ISO-accredited LDT (laboratory-developed test)., or a study specific Sofia RSV fluorescent immunoassay (SOFIA^®^RSV tests, Quidel) were used. The data in the manuscript has been modified to include the details

**Comment 3:** L105-106: what was your definition of LRTI?

**Response:** No protocol specific definition of LRTI was provided, the diagnosis of LRTI was done based on clinical decision. In general practice, LRTI clinical diagnosis is based on the presence of symptoms such as: respiratory distress (tachypnea or retraction and/or abnormalities on auscultation).

**Comment 4:** L99: Is a 2–7-day follow-up long enough to expect to observe factors associated with the length of hospital stay and the use of medical resources?

**Response:** Yes, we are convinced that in this study the follow-up period of 7 days is sufficient.

(1) All independent variables used in the modelling, on both length of hospital stay and the use of supplemental oxygen, use factors observed at baseline (day 1 in the hospital) namely demographic variables [age, sex], comorbidities [underlying risk], duration of symptoms before hospitalization, signs, and symptoms severity [individual and total PES3] and are thus not influenced by the length of study.
(2) We agree that stopping the follow-up period at day 7 could imply that the actual length of stay is not observed for patients who would be longer hospitalized. In this study, only 8/74 (10.8 %) subjects had a length of stay of longer than 7 days, and thus had a censored length of stay. The applied Cox proportional hazards model accounts for these censored observations.

For modelling of the length of hospital stay, results of the Cox proportional hazards model are now included in the Supplementary Materials Table S8. Results are qualitative similar as results of the logistic regression. This table has been mentioned in the section on ‘Factors affecting length of hospitalization’ in the manuscript.

**Comment 5:** Authors should clearly group together the potential risk factors for hospitalization and use of medical resources due to HRSV. That is to say socio-demographic factors, signs and symptoms, comorbidities, duration of symptoms before hospitalization, severity (PES, PES3).

**Response:** We agree with the reviewer. The text in the Statistical Analysis section (Page no 14/ line no 161) has changed to: “The following, at baseline available, independent variables (covariates) were considered: demographic variables [age, sex], comorbidities [underlying risk], duration of symptoms before hospitalization, signs and symptoms severity [individual PES3 items and total-PES3 score on day 1]. In addition, reception of oxygen supplementation on day 1 (Yes/No) was included as a covariate for the modelling of LOH. Three options were explored to incorporate the PES score on day 1: (1) the total PES score by summing the 8 individual components; (2) scores on the individual components of ability to feed, dyspnea and respiratory effort; and (3) the PES3 score. The results of options (2) and (3) are presented here.”

**Comment 6:** The authors must clarify the dependent variables, i.e. hospitalization (length of hospitalization) and use of medical resources (mechanical ventilation, oxygen supplementation, duration of oxygen supplementation, reception of supplementation oxygen on day 1, concomitant medication, doctor's visit for ARI before hospitalization).

**Response:** We agree with the reviewer. The text in the Statistical Analysis section (Page 14, line no 155) has been changed to: “Univariate and multivariate logistic regression models were applied to investigate whether covariates impact the dependent variables LOH (categorized as LOH ≤4 days and LOH >4 days) and the need for supplemental oxygen (subject received supplemental oxygen during hospitalization: Yes/No). Univariate and multivariate Cox proportional hazard regression models were implemented on the LOH (in days) and length of oxygen supplementation (in days).”

**Comment 7:** L140-143: this part should be presented in data analysis or socio-demographic data.

**Response:** Thanks for the suggestion, the text has now been moved to the Statistical analysis section

**Comment 8:** The authors should explain the orientation of the choice of the Wilcoxon test, chi square test, log rank and logistic regression.

**Response:** As mentioned in Statistical analysis section, all significance tests were 2-sided with a 5% significance level, where applicable. The ‘where applicable’ refers to the fact that chi-square test for independence of two categorical variables is always one-sided.

**Reviewer 2:**

The study demonstrates the issue well in disease severity and medical resource utilization of hospitalized RSV-positive children. Overall, this is a well-written manuscript and contributes valuable respiratory data in pediatric clinical practice. The methods are generally appropriate, although authors should clarify a few details and provide a rationale for using analytical methods to measure medical resource utilization parameters.

**Comment 1:**

*Introduction*

p.9, paragraph 3: Since the authors chose the age as a parameter for medical resource utilization categorized into 0-<3 months, 3-6 months, 6-<12 months, 12-<48 months, readers would want to see background epidemiological data of RSV in these age groups of children.

**Response:** The following text along with the corresponding references has been added in the introduction (Page 9, line no 69):

It has been documented that the clinical presentation of RSV infection in children differs according to age and may be influenced by the differences in their immune reaction to RSV. In a metareview, (Bont L et al., Defining the Epidemiology and Burden of Severe Respiratory Syncytial Virus Infection Among Infants and Children in Western Countries. Infect Dis Ther. 2016 Sep;5(3):271-98.) it was shown that the annual RSV hospitalization rates decreased with increasing age and varied by a factor of 2-3. Risks factors associated with RSV related medical resources utilization included: male sex; age <6 months; birth during the first half of the RSV season; crowding/siblings; and day-care exposure (high strength of evidence).

**Comment 2:** Methods

Study design

p.11, lines 93-95: Please explain on what grounds these hospitals were selected. Are these hospitals located in RSV endemic areas in the country?

**Response:** The study had a central site that coordinated the additional sites included. The hospitals were in Flanders, and through their locations allowed a good geographical coverage of the seasonal RSV circulation in that region. The study sites included tertiary academic center/university, large regional and small regional hospitals.

**Comment 3:** p.11, line 96: Since authors addressed epidemic seasons in the Introduction of the study, the S1 figure is unnecessary. Instead, please add study start and end dates for each respective year.

**Response:** We have added the study start and end date. The text now reads as:

This exploratory, prospective, multicenter study (NCT02133092) enrolled patients from clinical pediatric wards of four Belgian hospitals (one tertiary academic center and three regional hospitals: UZ Leuven, AZ Sint-Jan Brugge – Oostende campus Brugge, AZ Sint-Jan Brugge - Oostende campus Henri Serruys, and Heilig Hart Ziekenhuis Lier) during the 2013–2014 and 2014–2015 RSV epidemic seasons (Study initiated: 17 December 2013 and Study completed: 21 January 2015)

**Comment 4:** Study population

p.12, line 105: Please give more specifics of study subjects. Are all pediatric patients included who met inclusion criteria? Readers would want to know why the recruited subjects are only 75 when the RSV detection rate is almost 80% of children under five with severe acute respiratory infection in Belgium generally. (Subissi L, et al. Capturing respiratory syncytial virus season in Belgium using the influenza severe acute respiratory infection surveillance network, season 2018/19. Euro Surveill. 2020 Oct; 25(39): 1900627.)

**Response:** All pediatric patients included in the study met the inclusion criteria, however, not all patients that met the inclusion criteria were enrolled in the study. Furthermore, the study was not designed to measure RSV positivity rate in all patients hospitalized. The patients were not enrolled during the Weekends and public holidays.

One possible explanation for the difference between the positivity rate found in our research and the Subissi et al 2020 reference, could be different case definitions used for eligibility across the two studies, with any ARI for our study and SARI case definition for the referenced study.

We would like to highlight that in Belgium, similar to other countries, the positivity rate in surveillance data, are between 20-50%, depending on the intensity of RSV circulation, and aligned with our findings. (<https://www.tandfonline.com/doi/epub/10.1080/17843286.2018.1492509?needAccess=true>)

**Comment 5:** Demographics and clinical characteristics

p.12, line 118: Did authors consider including re-infection of RSV in underlying conditions?

**Response:** We have no information on whether the RSV event for which the child was included in the study is a possible re-infection of RSV. Indeed, this would have been valuable information, however, re-infections or prior hospitalization due to RSV were not captured.

**Comment 6:** Medical resource utilization

p.13, line 138: As indications of medical resource utilization might differ depending on the country or context, please specify indications for supplementary oxygen provided for patients involved in the study. Also, authors might need to consider this indication as one of the covariates in the analysis if the practice differs across hospitals. Supplementary oxygen is a highly vulnerable treatment depending on the capacity of the hospitals, especially in developing countries.

**Response:** The decision to provide O2 supplementation was done according to the Standard of care. Whereas there was no harmonization on O2 supplementation start and stop decisions, in general, considering that the participating sites were located in the same region /country, they likely had similar local guidelines for O2 supplementation, with 92% SpO2 used as a cut-off to guide hospitalization and request for O2 supplementation.

In this study we had 4 sites with, respectively, 14, 10, 24 and 27 subjects. Proportions of subjects receiving oxygen supplementation differs, markedly, by site with respectively 92.9% (13/14), 20.0% (2/10), 41.7% (10/24) and 70.4% (19/27). Further, it was observed that the site with highest proportions of oxygen supplementation also had the highest proportion of youngest subjects. However, as can be observed from the table below, overall, there is a trend in all sites that mainly the younger patients received supplemental oxygen.

|  | Received oxygen supplementation | | | | |
| --- | --- | --- | --- | --- | --- |
|  | All | Age,  0 - <3 months | Age,  3 - <6 months | Age,  6 - <12 months | Age,  12 - <48 months |
| Site 1 (n=14) | 13 (92.9 %) | 9 / 9 (100 %) | 1 / 2 (50.0 %) | - | 3 / 3 (100 %) |
| Site 2 (n=10) | 2 (20.0 %) | 1 / 2 (50.0 %) | 0 / 1 (0.0 %) | 0 / 2 (0.0 %) | 1 / 5 (20.0 %) |
| Site 3 (n=24) | 10 (41.7 %) | 4 / 6 (66.7 %) | 3 / 6 (50.0 %) | 1 / 4 (25.0 %) | 2 / 8 (25.0 %) |
| Site 4 (n=27) | 19 (70.4 %) | 9 / 11 (81.2 %) | 3 / 6 (50.0 %) | 6 / 7 (85.7 %) | 1 / 2 (50.0 %) |

Based on clinical input that an association exists between age and receiving supplemental oxygen and the results in the table above, it was decided not to include site as a covariate in the statistical models. Including site as a covariate could possibly dilute the effect of age. In addition, the manner the different sites recruited participants could be different (some sites could have included mainly sicker patients that have a higher probability of receiving supplemental oxygen). This difference is a potential factor for bias when site would be included as a covariate in the modelling.

This was also added to the Discussion section of the manuscript (see also below on the comment on potential sources of bias raised by the reviewer).

**Comment 7:** Statistical analysis

p.13, line 147 and line 152: Please briefly explain why Kaplan-Meier survival analysis and Cox proportional hazard regression models were performed with distinction. Please indicate all the variables included in each analysis respectively.

**Response:** Kaplan-Meier analysis can only be used for univariate analysis (thus one covariate at a time). Whereas the Cox proportional hazard regression model can incorporate multiple variables at the same moment.

We adjusted the Statistical Analysis section such that it is more clearly presented which variables are included in each analysis: “Univariate and multivariate logistic regression models were applied to investigate whether covariates impact the dependent variables LOH (categorized as LOH ≤4 days and LOH >4 days) and the need for supplemental oxygen (subject received supplemental oxygen during hospitalization: Yes/No). Univariate and multivariate Cox proportional hazard regression models were implemented on the LOH (in days) and length of oxygen supplementation (in days).

The following, at baseline available, independent variables (covariates) were considered: demographic variables [age, sex], comorbidities [underlying risk], duration of symptoms before hospitalization, signs and symptoms severity [individual PES3 items and total-PES3 score on day 1]. In addition, reception of oxygen supplementation on day 1 (Yes/No) was included as a covariate for the modelling of LOH. Three options were explored to incorporate the PES score on day 1: (1) the total PES score by summing the 8 individual components; (2) scores on the individual components of ability to feed, dyspnea and respiratory effort; and (3) the PES3 score. The results of options (2) and (3) are presented here.”

**Comment 8:** p. 14, line 163: Please explain why p-values without multiple comparison corrections were reported.

**Response:** It is common practice that multiple testing correction is not applied to multiple linear regression. We want to point to the following publication on multiple comparison [‘Adjust for Multiple Comparisons? It’s Not That Simple’ by A. Althouse, doi: https://doi.org/10. 1016/j.athoracsur.2015.11.024]. In our and the author’s opinion, the best approach is simply to (1) describe what was done in a study; (2) report point estimates, confidence intervals, and p-values; and (3) let readers use their own judgment about the relative weight of the conclusions. The author and others argue that because adjustment for multiple comparisons has several practical considerations that make the concept unreasonable to apply to every research paper, and particularly not to exploratory studies.

In confirmatory studies, which may lead to a change in clinical practice or approval of a new treatment, it is more important to guard against the possibility of false-positive results. When it comes to exploratory studies or post-hoc analysis of existing data, though, a strict adjustment for multiple comparisons is less critical, as long as the manuscript contains a clear statement acknowledging that.

**Comment 9:** Results: p. 15, line 170: Readers would want to know why children above 41 months did not take part in the study as inclusion criteria were pediatric patients up to 5 years old.

**Response:** Although the inclusion criteria were open to 5-year-old, most of the patients hospitalized were <1 year old; 41 months was the maximum observed age in the study. This is in alignment with what has been published in the field, with RSV burden of hospitalization been reported mainly in the very young children.

**Comment 11:** Table 1: Please clarify breastfeeding. Does it indicate breastfeeding at the point of the data collection?

**Response:** Yes, this was breastfeeding status at enrollment

**Comment 12:** Clinical disease kinetics

p.19, line 231-233: As shown in Fig 2, the ability to feed and wheezing were mentioned, but how about rales, rhonchi, or others as it appears to be presented more in patients with underlying risk factors too?

**Response:** Thank you for the suggestion, changes in rales and rochis and others have now been highlighted as well. Corresponding change in the text is as below

Higher scores i.e., worse condition in the ability to feed item for the 3–6 months group, wheezing; changes in rales and rochi, dyspnea, respiratory effort, and otitis for patients with underlying risk factors show that certain functions were affected more in certain groups.

**Comment 13:** Discussion

p.23, paragraph 4: In case authors do not have data on bacterial co-infection, it might look misguiding to state that 41.3% of the patients prescribed with antibiotics highlight overuse of antibiotics. If the authors have more explanation in this regard, please do so. Otherwise, please revise.

**Response:** - No data on bacterial co-infection was available. However, we based our statement on the following considerations:

- The frequency of RSV and bacterial co-infection in children is relatively low. Numerous studies have shown that the occurrence of a secondary or concurrent bacterial infection in hospitalized children with RSV lower respiratory tract disease is <1% (<https://adc.bmj.com/content/89/4/363?ijkey=04c663ca8ef80c49237de729d2677f9842cca96f&keytype2=tf_ipsecsha>)
- The over-use of antibiotics for treatment of RSV disease was highly documented in the field.

- [Reducing Antibiotic Use in Respiratory Syncytial Virus-A Quality Improvement Approach to Antimicrobial Stewardship - PubMed (nih.gov)](https://pubmed.ncbi.nlm.nih.gov/30229182/)
- [Antibiotic Overuse in Children with Respiratory Syncytial Virus Lower Respiratory Tract Infection - PubMed (nih.gov)](https://pubmed.ncbi.nlm.nih.gov/29601448/)

However, the following change in text (Page 23, line no 320) is made, to acknowledge the absence of bacterial co-infection data:

We did not collect data on bacterial co-infection; however, we suspect overuse of antibiotics based on the facts that numerous studies have shown that the occurrence of a secondary or concurrent bacterial infection in hospitalized children with RSV lower respiratory tract disease Nevertheless, frequency of RSV and bacterial co-infection in children is very low. The overuse of antibiotics for treatment of RSV disease has been documented before.

**Comment 14:** p.24, line 330: Please discuss more on the limitation of the study, including potential sources of bias.

**Response:** We have modified the limitations to identify the potential sources of bias. The limitations paragraph now reads as below:

Due to practical reasons, only a subset of patients admitted for RSV infections were enrolled in the study, therefore we cannot comment on the generalizability of the results. In addition, the way different sites recruited participants could be different (some sites could have included mainly sicker patients that have a higher probability of receiving supplemental oxygen). We realized that the patients included may not be representative for all RSV related hospitalizations in the participating sites. Since a strict protocol on initiation and discontinuation of oxygen supplementation was not implemented, all participating hospitals followed their in-house protocol, which may vary from each other. The participating hospitals also did not have documented discharge criteria. This difference is a potential factor for bias introduced by site effects. Although not excluded in the protocol, no intensive care cases were included, hence most severe cases were not captured.

**Comment 15: *Minor comments:***

Please correct typos such as:

p. 7, line 45: “PSE3” to “PES3”

p.8, line 59: “score” to “score.”

p.26, line 362: “children” to “children.”

**Response:** Thanks for noting. We have now corrected the typos.

_______________________________________
