# Supplementary material for: Risk factors for disease severity and increased medical resource utilization in respiratory syncytial virus (+) hospitalized children: A descriptive study conducted in four Belgian hospitals
Source: PLoS One. 2022 Jun 6;17(6):e0268532. doi: 10.1371/journal.pone.0268532 (PMC9170098; doi:10.1371/journal.pone.0268532)
Supplement: Attachment — Submitted filename: Response to Reviewers v2._PONE-D-21-25349_24Mar22.docx [file pone.0268532.s004.docx]

Journal Requirements:

Please review your reference list to ensure that it is complete and correct. If you have cited papers that have been retracted, please include the rationale for doing so in the manuscript text or remove these references and replace them with relevant current references. Any changes to the reference list should be mentioned in the rebuttal letter that accompanies your revised manuscript. If you need to cite a retracted article, indicate the article’s retracted status in the References list and also include a citation and full reference for the retraction notice.

[Note: HTML markup is below. Please do not edit.]

Reviewers' comments:

Reviewer's Responses to Questions

**Comments to the Author**

1. If the authors have adequately addressed your comments raised in a previous round of review and you feel that this manuscript is now acceptable for publication, you may indicate that here to bypass the “Comments to the Author” section, enter your conflict of interest statement in the “Confidential to Editor” section, and submit your "Accept" recommendation.

Reviewer #1: (No Response)

Reviewer #2: All comments have been addressed

2. Is the manuscript technically sound, and do the data support the conclusions?

The manuscript must describe a technically sound piece of scientific research with data that supports the conclusions. Experiments must have been conducted rigorously, with appropriate controls, replication, and sample sizes. The conclusions must be drawn appropriately based on the data presented.

Reviewer #1: Yes

Reviewer #2: Yes

3. Has the statistical analysis been performed appropriately and rigorously?

Reviewer #1: Yes

Reviewer #2: Yes

4. Have the authors made all data underlying the findings in their manuscript fully available?

The [PLOS Data policy](http://www.plosone.org/static/policies.action#sharing) requires authors to make all data underlying the findings described in their manuscript fully available without restriction, with rare exception (please refer to the Data Availability Statement in the manuscript PDF file). The data should be provided as part of the manuscript or its supporting information, or deposited to a public repository. For example, in addition to summary statistics, the data points behind means, medians and variance measures should be available. If there are restrictions on publicly sharing data—e.g. participant privacy or use of data from a third party—those must be specified.

Reviewer #1: Yes

Reviewer #2: Yes

5. Is the manuscript presented in an intelligible fashion and written in standard English?

PLOS ONE does not copyedit accepted manuscripts, so the language in submitted articles must be clear, correct, and unambiguous. Any typographical or grammatical errors should be corrected at revision, so please note any specific errors here.

Reviewer #1: Yes

Reviewer #2: Yes

6. Review Comments to the Author

Please use the space provided to explain your answers to the questions above. You may also include additional comments for the author, including concerns about dual publication, research ethics, or publication ethics. (Please upload your review as an attachment if it exceeds 20,000 characters)

Reviewer #1: Comments to the Author
Thanks to the authors for responding to the questions raised. There are, however, the questions below that I would like further clarification.

Methodology
L97: In general, it is preferred to describe the following information: (company name, City, State. Country) for company information of the experimental kit used.
Thank you for modifying the document in accordance with the comment above. However, it will be better to also specify in the main manuscript that the qRT-PCR assay was homemade.

**Response:** Thank you for your suggestion, we have specified that qRT-PCR assay was homemade in the manuscript.

L99: Is a 2–7-day follow-up long enough to expect to observe factors associated with the length of hospital stay and the use of medical resources?
Thank you for providing the explanations in the letter in relation to the comment above. You affirm that you are convinced that 7 days is long enough to observe all the outcomes of the use of health resources. It would be greatly appreciated if you provide a reference that supports your statement. Also, the explanation that 8/74 participants had more than 7 days in hospital would be important to mention in the main manuscript.

**Response:** Thank you for your suggestion, we already have this statement in the result section of the manuscript (L225, hospitalization paragraph of result section). We have now added the additional explanation along with the references that would support the length of hospital stay we used in this study.

1) The objective of the study was to describe clinical and medical burden during the hospitalization. However, most of the children are discharged with ongoing signs/symptoms that may drive additional medical resources utilization after hospital discharge. Due to the study design, we could not capture the MRUs after discharge, this being another limitation of the study. We have included this in the discussion section
2) We consider that 7 days follow-up is sufficient to monitor the use of hospital health resources in the enrolled population, besides only 8/74 participants enrolled were hospitalized for more than 7 days. We have also included some literature in the discussion section to support the length of the hospital stay observed in our study,[median of 5.0 days (2–7) d CI], which is similar to the length of hospitalization observed in a Belgium study (LOH: median 5 days, 3-11 CI) ([Subissi et al.,](https://pubmed.ncbi.nlm.nih.gov/33006303/) *[Euro Surveill](https://pubmed.ncbi.nlm.nih.gov/33006303/)*[. 2020 Oct 1; 25(39](https://pubmed.ncbi.nlm.nih.gov/33006303/)): 1900627) and in Spain (LOH: mean 4.8 +/- 11d) ([Viguria et al., PLoS One. 2018 Nov 15;13(11):e0206474](https://pubmed.ncbi.nlm.nih.gov/30439987/))
3) The logistic regression analysis on the probability of hospitalization length (LOH ≤ 4 days, LOH > 4 days) and probability of requiring oxygen supplementation, as well as the Cox regression models on the length of hospitalization and length of oxygen supplementation only have baseline covariates included. Thus, the included covariates are independent on the length of follow-up of the patients. The applied Cox proportional hazards model account for censored observations, and thus all available information of the 8 patients with more than 7 days of hospitalizations is included.

Reviewer #2: The authors have clarified all of the questions I raised in my previous review except one problem.
Also, the two copies of the manuscript provided were somewhat different. I would suggest using a file named “Observe001_MSS_Resubmission version_clean copy” instead of a file named “Observe001_MSS_PLOS ONE” as the latter does not seem to reflect all the revisions made.
In general, the paper appears to be worthwhile, and I would accept after addressing the following issue:

**Response:** Thank you for your suggestion and accepting the paper, we have renamed the file to, ‘Observe001_MSS_Resubmission version_clean copy’ as suggested. We have also verified and checked if the current version reflects all the revisions, and we are able to see the revisions.

Table 1: Please add and revise the appropriate term for the “breastfeeding” variable. Although the authors stated that the variable shows breastfeeding status at enrolment, how about older children who had stopped breastfeeding as your study subjects are up to 41 months old? If you marked them as “no” in the breastfeeding variable, that would be a major concern. Showing the breastfeeding duration is vital as a sufficient breastfeeding period can provide a long-term protective effect against respiratory tract infections. Authors might also need to include breastfeeding as one of the independent variables in multivariate analysis. Because it is well reported that breastfeeding is significant in reducing the rate of severe RSV infection cases. (Please refer to <https://www.thelancet.com/series/breastfeeding>)

**Response:** Thank you for suggestion and valuable input regarding the breastfeeding being significant in reducing the rate of severe RSV infection cases.

We have changed the term to ‘currently breastfed’ in the manuscript. Unfortunately, we don’t have information on breastfeeding status or length in the past. We added in the discussion that it is a limitation that we cannot study the possible long-term protective effect of breastfeeding on hospitalization characteristics.

The relevant literature published in this field assessed breastfeeding as a risk factor for progression to hospitalization. However, studies that assessed breastfeeding as a risk factor for severity of disease and length of hospitalization and MRU during hospitalization are limited. Below are some relevant references

1) This study suggests that there was not a significant association between breastfeeding and bronchiolitis severity score or length of hospital stay ([Vereen S, Gebretsadik T, Hartert TV et al., *Pediatr Infect Dis J.* 2014 Sep;33(9):986-8)](https://pubmed.ncbi.nlm.nih.gov/24751863/).

2) This article states breastfeeding (<2months or not) was a predictor of progression to hospitalization ([Blanken MO, Koffijberg H, Nibbelke EE et al.,](https://pubmed.ncbi.nlm.nih.gov/23554987/) *[PLoS One.](https://pubmed.ncbi.nlm.nih.gov/23554987/)* [2013;8(3):e59161](https://pubmed.ncbi.nlm.nih.gov/23554987/))

3) In a meta-analysis, breastfeeding was observed to be associated with RSV ALRI. However, the definition used for breastfeeding in the reviewed articles was different - No breastfeeding = [no breastfeeding for first 14 days, <3 months breastfeeding or lack of exclusive breastfeeding] ([Shi T, Balsells E, Wastnedge E, Singleton R et al., *J Glob Health.* 2015 Dec;5(2):020416](https://pubmed.ncbi.nlm.nih.gov/26682048/))

Current breastfeeding status (yes/no) is associated with age (see Table S3) with higher proportions of currently being breastfed in the youngest age group.

A Kaplan-Meier analysis showed no differences between current breastfeeding status and length of hospital stay (p=0.55). We also included current breastfeeding status as one of the independent variables in both a univariate and the multivariate regression analyses for length of hospital stay. Current breastfeeding status showed no differences in these regression models.

In our study, patients with current breastfeeding have a longer length of oxygen supplementation (Kaplan-Meier analysis, p=0.046). This result, however, is confounded with age. A univariate logistic regression analysis for the probability of receiving oxygen supplementation shows a significant effect of breastfeeding status. Patients receiving currently breastfeeding have a higher probability of receiving oxygen with OR=3.33 [1.18 – 10.51]. However, in a multivariate logistic regression (and thus corrected for age) no effect is observed anymore of current breastfeeding status (OR = 0.73 [0.15 – 3.40]). Similarly, in the multivariate Cox regression model no significant effect of breastfeeding status is observed (HR = 1.19 [0.64 – 2.22]).

7. PLOS authors have the option to publish the peer review history of their article ([what does this mean?](https://journals.plos.org/plosone/s/editorial-and-peer-review-process#loc-peer-review-history)). If published, this will include your full peer review and any attached files.


If you choose “no”, your identity will remain anonymous but your review may still be made public.


**Do you want your identity to be public for this peer review?** For information about this choice, including consent withdrawal, please see our [Privacy Policy](https://www.plos.org/privacy-policy).

Reviewer #1: No

Reviewer #2: **Yes:** Amarjargal Dagvadorj, MD, MSc, DrPH

[NOTE: If reviewer comments were submitted as an attachment file, they will be attached to this email and accessible via the submission site. Please log into your account, locate the manuscript record, and check for the action link "View Attachments". If this link does not appear, there are no attachment files.]

While revising your submission, please upload your figure files to the Preflight Analysis and Conversion Engine (PACE) digital diagnostic tool, <https://pacev2.apexcovantage.com/>. PACE helps ensure that figures meet PLOS requirements. To use PACE, you must first register as a user. Registration is free. Then, login and navigate to the UPLOAD tab, where you will find detailed instructions on how to use the tool. If you encounter any issues or have any questions when using PACE, please email PLOS at [figures@plos.org](mailto:figures@plos.org). Please note that Supporting Information files do not need this step.
